# The first case of combined pancreatic neuroendocrine tumour and neuroendocrine carcinoma

**DOI:** 10.1093/jscr/rjae746

**Published:** 2024-12-02

**Authors:** Tegan Lun, Christophe Rosty, Pranavan Palamuthusingam

**Affiliations:** Department of Surgery, Townsville University Hospital, Townsville, 4814, QLD, Australia; Envoi Specialist Pathologists, Brisbane, 4059, QLD, Australia; The University of Queensland, Brisbane, 4072, QLD, Australia; Department of Surgery, Royal Brisbane and Women’s Hospital, Brisbane, 4006, QLD, Australia; Department of Surgery, The Wesley Hospital, Brisbane, 4066, QLD, Australia

## Abstract

Pancreatic neuroendocrine neoplasms are currently thought to originate from distinct progenitor cells that cannot differentiate into each other. We present the first reported case of a combined pancreatic neuroendocrine tumour and neuroendocrine carcinoma in a 58-year-old man who was investigated for abdominal pain and constipation. Imaging revealed a large left upper quadrant mass infiltrating the pancreatic body and tail, splenic hilum, and posterior stomach wall, with five hepatic metastases. This was treated with neoadjuvant and adjuvant chemotherapy, debulking surgery, and lutetium-177-DOTATATE peptide receptor radionuclide therapy. This case emphasises the importance of molecular imaging, meticulous microscopic examination, and multidisciplinary discussion for accurate diagnoses, improved prognostication, and efficacious treatment.

## Introduction

Pancreatic neuroendocrine neoplasms (panNENs) were recognised by the 2017 WHO classification system and eighth edition of the AJCC Cancer Staging Manual as having two distinct types with varied histopathologic criteria: pancreatic neuroendocrine tumours (panNETs) and pancreatic neuroendocrine carcinomas (panNECs) [1, 2]. PanNETs are well differentiated, low-proliferating, and graded by proliferation index. PanNECs are high grade by definition and poorly differentiated, resulting in an aggressive phenotype and poor prognosis. However, knowledge of the differences in panNEN origin, treatment response, and prognosis is still evolving. We present the first reported case of a combined panNET and panNEC, which was treated with neoadjuvant and adjuvant chemotherapy, debulking surgery, and lutetium-177-DOTATATE peptide receptor radionuclide therapy (Lu-PRRT).

## Case report

**Figure 1:**
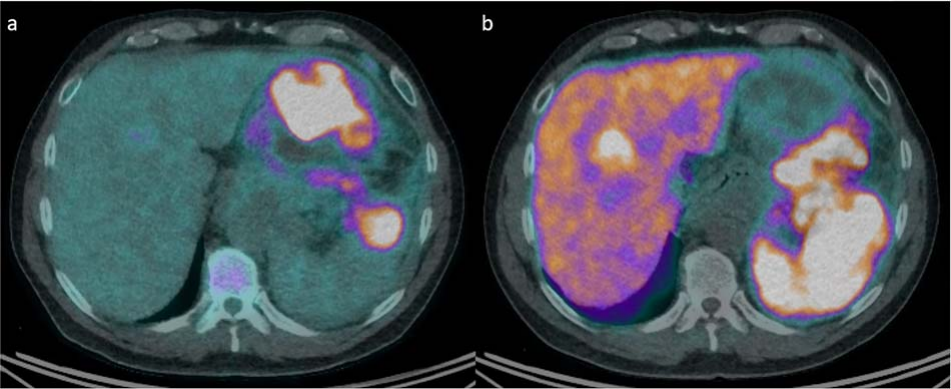
FDG and DOTATATE PET/CT of the mass. (a) Intensely FDG-avid mass with patchy regions of relative photopenia and necrosis. (b) Heterogenic DOTATATE uptake with poor uptake in the FDG avid components at the inferior aspect of the pancreatic mass and superior-posteriorly to the stomach.

An otherwise well 58-year-old man presented to our emergency department with 1 week of intermittent upper abdominal pain and constipation. Examination revealed mild distension and upper abdominal tenderness. Laboratory investigations showed a leukocytosis 18 × 10^9^/L, serum chromogranin A of 750 ng/ml, and CA19.9 of 79 U/ml. Computed tomography (CT) showed a 21 × 12 × 13 cm left upper quadrant mass, which infiltrated the pancreatic body and tail, splenic hilum, and posterior stomach wall, with five hepatic metastases. Liver biopsy demonstrated grade 2 panNET. Both fluorodeoxyglucose (FDG) and DOTATATE positron emission tomography (PET)/CT were performed (Fig. 1). The mass was intensely FDG-avid, with areas of necrosis and mediastinal lymph node uptake. There was corresponding poor DOTATATE uptake in the FDG avid components, which suggested more aggressive undifferentiated tumour.

**Figure 2:**
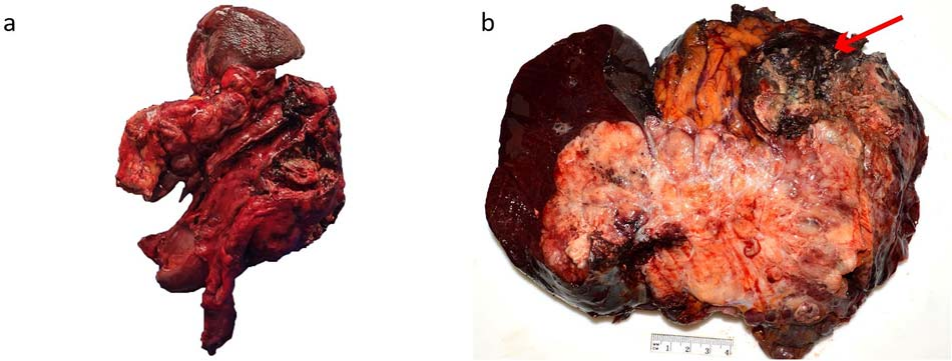
Macroscopic appearance of the tumour. (a) Distal pancreatectomy, splenectomy, partial gastrectomy, and splenic flexure specimen. (b) 190 × 170 mm solid lesion mostly cream and yellow with a hemorrhagic and necrotic nodule (arrow).

**Figure 3:**
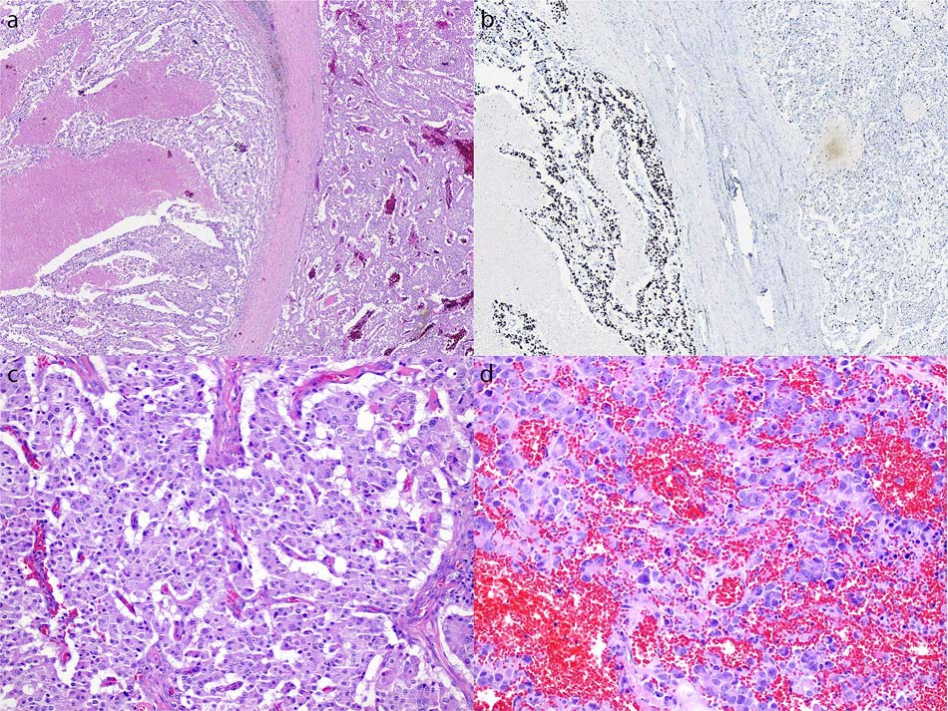
Microscopic appearance of the tumour. (a) Hematoxylin and eosin (H&E) stain at 20× magnification of both the NET (right) and NEC (left) components. (b) Ki-67 immunostain at 20× magnification of both the NET and NEC components. (c) H&E stain at 200× magnification showing well differentiated NET with mild cytological atypia and low mitotic activity. (d) H&E stain at 200× magnification showing NEC with large pleomorphic cells and high mitotic rate.

The patient commenced capecitabine/temozolomide—a first-line therapy for panNET. After 1 month, they developed sepsis requiring intensive care admission for vasopressor support. Repeat CT demonstrated a fistulous communication between the transverse colon and tumour cavity. Subsequently, a second cycle of chemotherapy was commenced using carboplatin/etoposide before a distal pancreatectomy, splenectomy, partial gastrectomy, and splenic flexure resection (Fig. 2) for debulking. Histopathology showed a 19 cm T3N1M1 panNEN clear of margins, but with lymphatic invasion (1/8) (Fig. 3). The predominant component was a grade 3 panNET characterised by mild cytological atypia, 22% Ki-67 proliferation rate, and splenic and gastric wall infiltration. The second component consisted of a large cell panNEC with poor differentiation, marked nuclear atypia, >95% Ki-67 proliferation rate, necrosis, and colonic wall infiltration. Immunohistochemistry was positive for chromogranin and synaptophysin but negative for trypsin, excluding an acinar cell carcinoma.

The postoperative course was complicated by a left sub-phrenic abscess necessitating pigtail stent insertion. With stable residual disease superior to the left kidney on re-staging PET, the patient received two more cycles of carboplatin/etoposide with partial response followed by disease progression. Two cycles of Lu-PRRT were then associated with a response to DOTATATE avid disease, but significant progression of FDG avid disease. At 12-months post-operatively, the patient had been referred for consideration of SIRTEX treatment.

## Discussion

PanNENs are known to originate from precursor cells in the pancreatic ductal epithelium with neuroendocrine differentiation; however, understanding of their pathogenesis is evolving. PanNETs and panNECs are currently thought to originate from different progenitor cells that cannot differentiate into each other. For instance, studies indicate an endocrine descent of panNETs with mutations in genes such as *MEN1*, *ATRX*, and *DAXX* [3]. In contrast, DNA methylation analysis and immunohistochemistry have demonstrated a potential exocrine origin for panNECs with a mutational profile similar to pancreatic ductal adenocarcinomas [4]. Genetic testing has also facilitated both the distinction between panNENs, which overlap in mitoses and Ki-67 index, and the identification of therapeutic targets. Additionally, the differing genetic risk factors and panNEN rarity would suggest that combined panNET and panNEC is likely a sporadic phenomenon.

Molecular imaging can also delineate panNENs for treatment and prognostication. For instance, DOTATATE PET/CT has a sensitivity of 80% for panNETs, but only 40% for panNECs due to the lack of differentiation and somatostatin receptors. In contrast, FDG PET/CT is more sensitive for NECs, with uptake rates of up to 100% for panNECs and 50% for panNETs [5]. Baseline SUV_max_ in both imaging modalities has also been used to predict response and progression-free survival following Lu-PRRT [6, 7]. Imaging was particularly useful in this case for suggesting an aggressive undifferentiated component, and directing treatment type and duration.

There is no consensus on the management of synchronous panNENs, with differing therapeutic recommendations for each subtype. The role of upfront palliative resection of non-functioning panNENs is debated due to the infrequency of tumour-related symptoms; however, debulking surgery is recommended for alleviating symptoms relating to tumour burden or carcinoid syndrome [8, 9]. For advanced panNETs, temozolomide-based therapies are recommended for overall survival benefit [8, 10]. In contrast, platin-based regimens with etoposide or irinotecan are recommended for panNECs, with short-term effects and no established second-line therapy [8, 11]. Additionally, phase II and III clinical trials using Lu-PRRT for gastroenteropancreatic NENs have shown encouraging survival rates, radiographic regression, and improvement in quality of life, despite a 4% risk of persistent haematological toxicity [12–15]. Our patient received debulking surgery for symptomatic relief, four cycles of chemotherapy with a focus on treating the aggressive component after tissue diagnosis, and Lu-PRRT given the risk of aggressive progression and recurrence.

## Conclusion

Growing insight into panNEN pathogenesis has led to improved staging paradigms, treatment, and prognostication; however, there is a need for ongoing research to further our understanding and patient outcomes. Ultimately, this case emphasizes the importance of molecular imaging, meticulous microscopic examination, and multidisciplinary discussion for accurate diagnoses, improved prognostication, and efficacious treatment.
